# An Improved Path-Finding Method for the Tracking of Centerlines of Tortuous Internal Carotid Arteries in MR Angiography

**DOI:** 10.3390/jimaging10030058

**Published:** 2024-02-28

**Authors:** Se-On Kim, Yoon-Chul Kim

**Affiliations:** Division of Digital Healthcare, College of Software and Digital Healthcare Convergence, Yonsei University, Wonju 26493, Republic of Korea; kso323@yonsei.ac.kr

**Keywords:** cerebral arteries, path finding, image segmentation, intracranial arteries, magnetic resonance angiography, blood vessel

## Abstract

Centerline tracking is useful in performing segmental analysis of vessel tortuosity in angiography data. However, a highly tortuous) artery can produce multiple centerlines due to over-segmentation of the artery, resulting in inaccurate path-finding results when using the shortest path-finding algorithm. In this study, the internal carotid arteries (ICAs) from three-dimensional (3D) time-of-flight magnetic resonance angiography (TOF MRA) data were used to demonstrate the effectiveness of a new path-finding method. The method is based on a series of depth-first searches (DFSs) with randomly different orders of neighborhood searches and produces an appropriate path connecting the two endpoints in the ICAs. It was compared with three existing methods which were (a) DFS with a sequential order of neighborhood search, (b) Dijkstra algorithm, and (c) A* algorithm. The path-finding accuracy was evaluated by counting the number of successful paths. The method resulted in an accuracy of 95.8%, outperforming the three existing methods. In conclusion, the proposed method has been shown to be more suitable as a path-finding procedure than the existing methods, particularly in cases where there is more than one centerline resulting from over-segmentation of a highly tortuous artery.

## 1. Introduction

Cerebrovascular imaging such as X-ray, computed tomography (CT), and magnetic resonance imaging (MRI) can non-invasively provide morphometric information on cerebral vessels. The detection of arteries’ centerlines is essential for the extraction of geometric information of the cerebral arteries [1,2,3], such as their tortuosity, thickness, and spatial variations [4,5,6]. It is related to the detection of bifurcations of interest for the labeling of the intracranial arteries [7,8,9]. Recently, the centerlines have been used as input to a topology-aware graph network model to perform vessel labeling in head and neck CT angiography [10]. Numerous research studies have associated the morphometric characteristics of the cerebral arteries with cerebrovascular diseases such as atherosclerosis and aneurysm [11,12,13,14,15].

**Figure 1:**
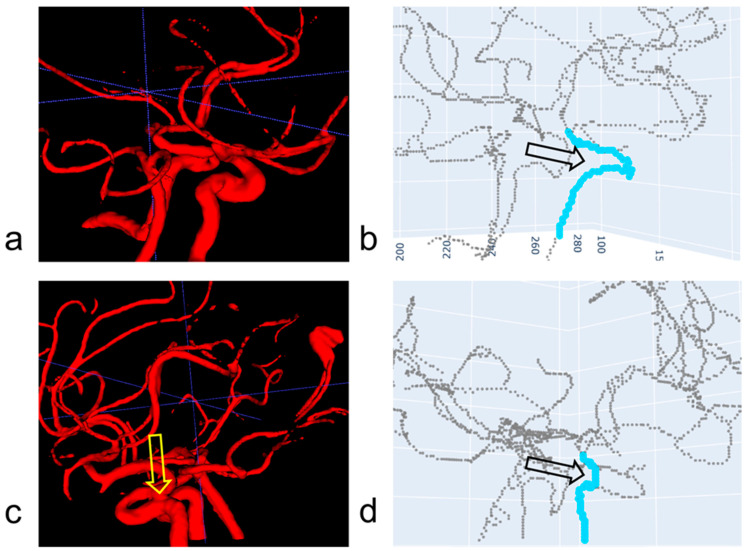
Examples of path-finding results in the ICA. (**a**) Subject A’s segmented artery. (**b**) Subject A’s skeleton and a path found using Dijkstra algorithm. The found path is indicated by the light blue line in (**b**) and is correct. (**c**) Subject B’s segmented artery. The segmented ICA is over-segmented such that the two segmented arterial portions are joined (see the yellow hollow arrow). (**d**) Subjects B’s skeleton and a path found using Dijkstra algorithm. The found path is indicated by the light blue line in (**d**) and is incorrect. Two centerlines are formed as indicated by the black hollow arrow in (**d**) after the skeletonization of the artery in (**c**). Hence, it was necessary to develop a new method to correctly identify the path along the ICA in (**d**).

Path-finding algorithms have been used to automatically extract the centerlines of arteries [16,17,18,19]. The Dijkstra and A* algorithms are well known for their effectiveness in finding the shortest path between two endpoints of an artery’s centerline [16]. A recent experimental study has indicated that these two algorithms are accurate in most arterial segments in the circle of Willis, except for the internal carotid arteries (ICAs) [17]. ICAs are inherently tortuous at the level of C4–C7 [20]. Due to the high level of tortuosity in the ICAs, they can be over-segmented. A similar study with CT angiography pointed out the presence of artificially created vessels or artificial loops resulting from segmentation errors [21]. They were referred to as shortcuts, which cause problems with the shortest path-finding algorithms. After a skeletonization process, more than one centerline can occur in an ICA segment. Hence, when the shortest path-finding process is performed given two endpoints where one lies in the C4 and the other lies in the C7, it can result in an erroneous path, and often a path whose length is shorter than the length of the correct path. Figure 1 illustrates a case where the shortest path-finding algorithm can lead to correct and incorrect path-finding results (compare Figure 1b and Figure 1d). The results depend on the absence or presence of a spurious centerline. A spurious centerline is indicated by the black hollow arrow in Figure 1d.

In this study, we propose a new method to address the issue of inaccurate path-finding results for the labeling of the ICA segment. The proposed method consists of a series of depth-first search (DFS) algorithms, where a DFS-based path finding is performed after changing the order of visiting of the 26-neighborhood voxels. The order was randomized via random shuffling. The repetition of random shuffling would produce a variety of possible paths connecting the two endpoints. As the next step, the proposed method finds the most appropriate ICA paths out of all the possible realizations of the paths. The proposed path-finding algorithm is compared to three existing path-finding methods in ICAs with regard to accuracy.

## 2. Materials and Methods

### 2.1. Data and Preprocessing

The publicly available time-of-flight magnetic resonance angiography (TOF MRA) image data from the IXI dataset (https://brain-development.org/ixi-dataset, accessed on 27 February 2024) were used for this study. Sixty subjects’ data were considered to evaluate the performance of path-finding algorithms in the ICA segments. Quite a few subjects’ data had the problem of incorrect path findings in the ICAs when the Dijkstra and A* algorithms were used [17]. This motivated us to develop a new method in order to improve the accuracy of the path-finding procedure in the ICA segments. A bicubic interpolation along the slice dimension was performed to generate isotropic resolution image data with the same voxel spacing of 0.6 mm in all three dimensions. A three-dimensional (3D) seeded region-growing algorithm was used to segment the arteries [22]. The segmented binary mask of the arteries underwent a skeletonization procedure [23] in order to obtain centerlines of the arteries.

### 2.2. Proposed Method

**Figure 2:**
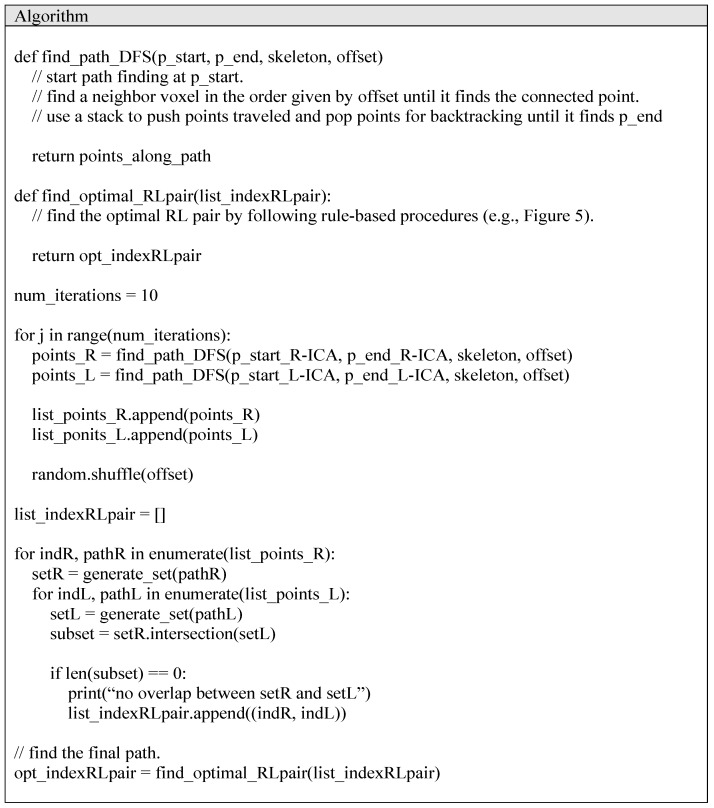
Pseudocode of the proposed path-finding algorithm.

**Figure 3:**
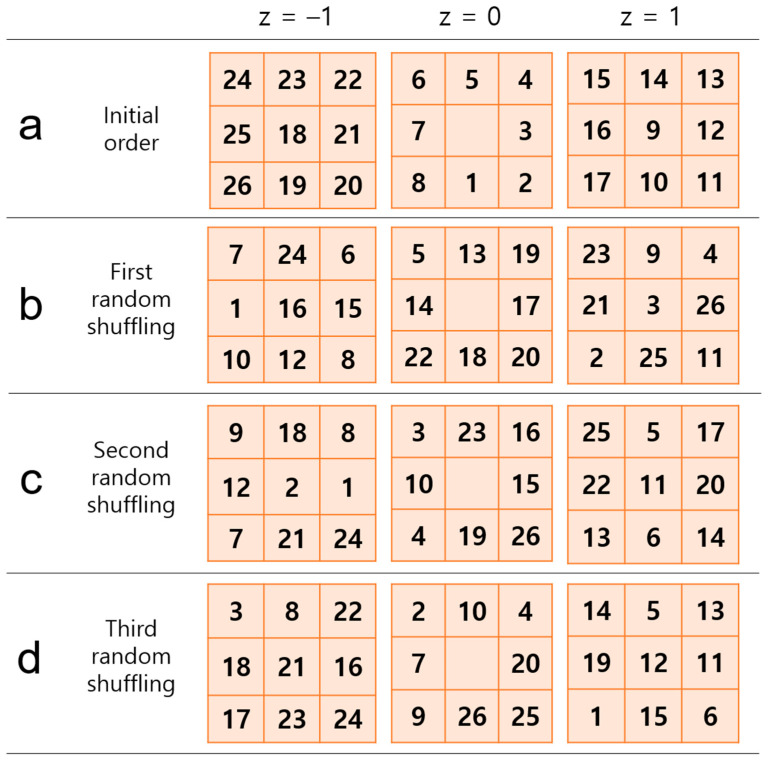
Variation in the order of visiting of neighboring voxels after random shuffling of the indices of the directions. Each row has its own order for the neighboring voxel search in the 26-connected neighborhood when the depth-first search (DFS) algorithm is used to find a path in 3D. The z at the top indicates a slice offset relative to the current voxel of interest, which has no number in the center at the z = 0 level. The number in each cell represents the order of visiting neighboring voxels. The random shuffling results in a randomized ordering in the 26-connected neighborhood. (**a**) Conventional sequential search order used in a typical DFS algorithm in 3D. (**b**–**d**) The first three random shuffling results. The selection of the voxel-visiting order affects the path-finding result.

**Figure 4:**
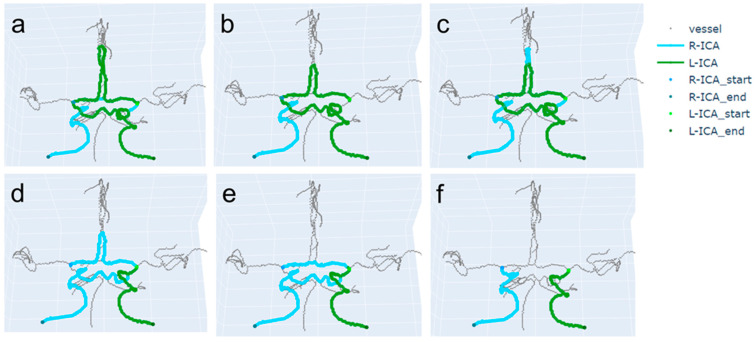
A variety of paths found when using the DFS-based path-finding algorithm with different orders of visiting of the neighborhood voxels due to random shuffling of the order. (**a**–**e**) Incorrect path-finding results with detoured paths. The R-ICA path overlaps with the L-ICA path in (**a**–**e**). These overlapped cases are discarded when selecting the correct paths. (**f**) Paths that do not overlap with respect to each other.

**Figure 5:**
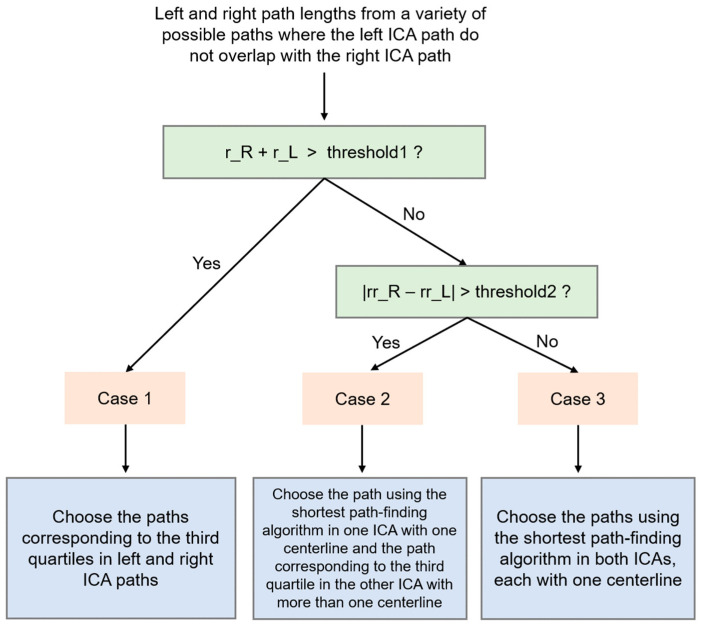
A flowchart for selecting an appropriate path among all possible paths in the left and right ICA centerlines. r_R (or r_L) refers to the range of the path lengths in the right ICA (or the left ICA). rr_R (or rr_L) refers to the relative range of the path lengths in the right ICA (or the left ICA).

The pseudocode of the proposed path-finding algorithm is shown in Figure 2. After a random shuffling process, the neighborhood search order can vary, as shown in Figure 3. The proposed method repeats random shuffling to generate a variety of path-finding results. In this study, we set the number of iterations to 10. First, the method discards cases with any overlap between the path found from the left ICA and the path found from the right ICA. The overlap can occur when a left (or right) ICA’s path is detoured such that it meets a right (or left) ICA’s path (Figure 4a–e). Second, the method selects an appropriate path based on the analysis of histograms of the right and left ICA path lengths. A flowchart for finding appropriate paths is illustrated in Figure 5. The path-finding process first identifies one of the three cases and then applies an appropriate path selection procedure according to the rule described in Figure 5.

#### 2.2.1. Case 1—Detection of a Case Where There Are Two ICA Segments with More than One Centerline

**Figure 6:**
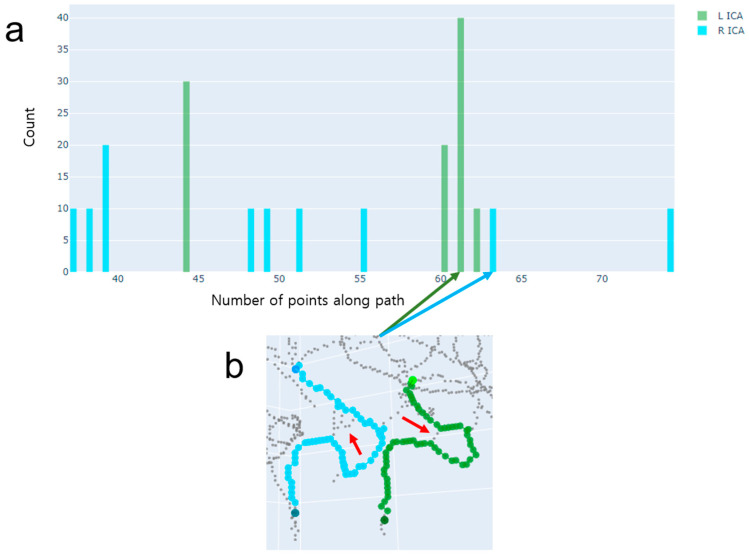
An example of Case 1, where there are two ICA segments with more than one centerline. (**a**) A histogram of path lengths. A path length is defined as the number of voxel locations along the path when using the DFS path-finding algorithm. The histogram represents a distribution of path lengths separately for the left ICA (green) and right ICA (cyan). (**b**) The final paths chosen by the proposed method. These paths have been correctly chosen. Note that the numbers of points belonged to the third quartiles in the left and right ICAs. The shortest path-finding algorithm would select paths that include the spurious centerlines indicated by the red arrows.

Because there are two ICA segments with more than one centerline, the range of path lengths is larger than in the other two cases, which have at most one ICA segment with more than one centerline. We developed a selection rule for Case 1, as shown in Figure 5. We selected Case 1 if the sum of ranges of the left and right ICA path lengths was greater than a threshold value. In our study, we empirically chose the threshold value of 40. The histograms of left and right ICAs tend to produce distributions that are similar to each other (Figure 6). Since there are shortcuts that have short path lengths, it is appropriate to find a path whose path length is in the third quartile.

#### 2.2.2. Case 2—Detection of a Case Where There Is Only One ICA Segment with More than One Centerline

**Figure 7:**
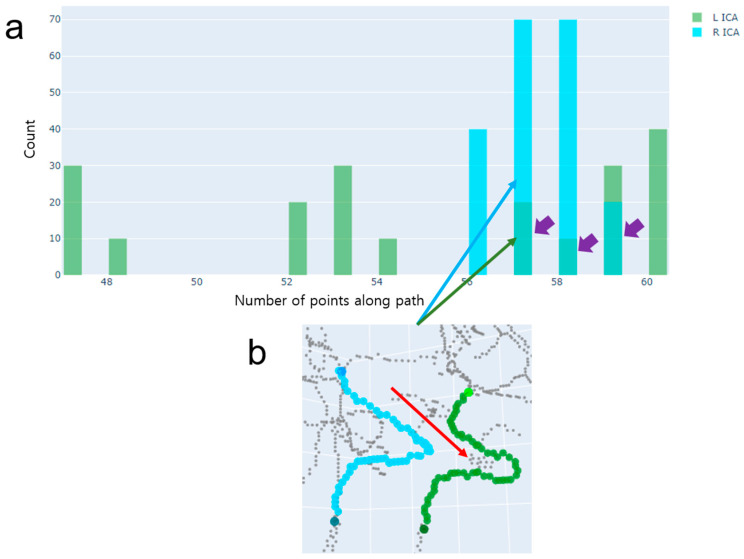
An example of Case 2, where there is only one ICA segment with more than one centerline. (**a**) A histogram of path lengths. A path length is defined as the number of voxel locations along the path when using the DFS path-finding algorithm. The histogram represents a distribution of path lengths separately for the left ICA (green) and right ICA (cyan). Note that the different shades (purple arrows) of green and cyan indicate overlap of the histograms of the left and right ICAs. (**b**) The final paths chosen by the proposed method. These paths have been correctly chosen. Note that the median path length was chosen for the right ICA and the third quartile path length was chosen for the left ICA. The shortest path-finding algorithm would select a path that includes the spurious centerlines indicated by the red arrow.

The histograms of left and right ICAs tend to produce distributions which are very different from each other (Figure 7). We selected Case 2 if the sum of ranges of left and right ICA path lengths was less than the range threshold value of 40, and the difference between the relative ranges of left and right ICA path lengths was greater than the relative range threshold value (Figure 5). In our study, we empirically chose the threshold value of 0.015. We defined the relative range, as shown in Equation (1).

(1) $$Relativerange\left(rr\right)=\frac{\max\left(path\_len\right)-\min\left(path\_len\right)}{median\left(path\_len\right)},$$

where *path_len* is the path lengths, each of which was calculated from a path found by the DFS path-finding algorithm with an order of visiting neighboring voxels via random shuffling. For simplicity, the path length was defined as the number of voxel locations that form the path as a result of the output of the DFS algorithm. Since one ICA has shortcuts that have short path lengths resulting from a series of DFSs with random shuffling, it is appropriate to find a path whose path length is in the third quartile. Since the other ICA has no shortcut, it is appropriate to find a path using the shortest path-finding algorithm (i.e., Dijkstra algorithm).

#### 2.2.3. Case 3—Detection of a Case Where There Is No ICA Segment with More than One Centerline

**Figure 8:**
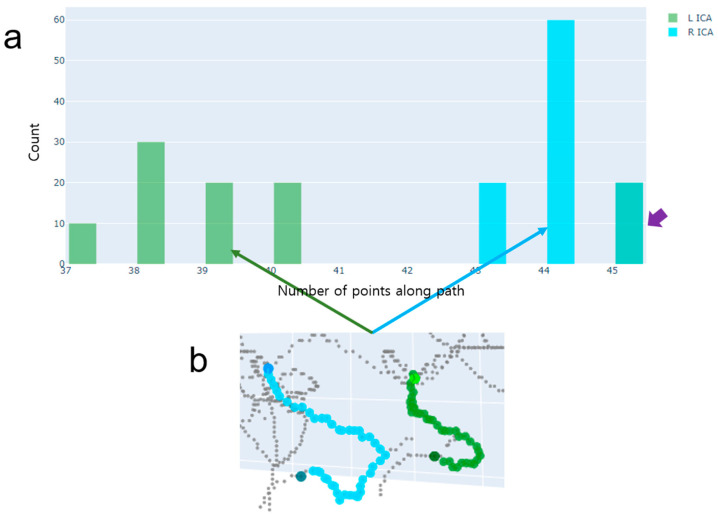
An example of Case 3, where there is no ICA segment with more than one centerline. (**a**) A histogram of path lengths. A path length is defined as the number of voxel locations along the path when using the DFS path-finding algorithm. The histogram represents a distribution of path lengths separately for the left ICA (green) and right ICA (cyan). Note that the purple arrow indicates overlap of the histograms of the left and right ICAs. (**b**) The final paths chosen by the proposed method. These paths have been correctly chosen.

Like Case 1, the histograms of left and right ICAs tend to produce distributions that are similar to each other (Figure 8). However, the distributions would be concentrated around a certain peak and yield less variations than Case 1. Hence, the differentiation between Case 2 and Case 3 can be made based on the difference between the relative ranges of left and right ICA path lengths. Since both have no shortcuts resulting from spurious centerlines, it is appropriate to find paths using the shortest path-finding algorithm (i.e., the Dijkstra algorithm).

### 2.3. Evaluation

We evaluated four path-finding methods in terms of path-finding accuracy: (Method 1) DFS algorithm, (Method 2) Dijkstra algorithm, (Method 3) A* algorithm, and (Method 4) the proposed algorithm. The pseudocode of Method 1 is shown in Appendix A. In this study, all the methods were implemented in Python. Method 1 is a simple path-finding algorithm that is based on a pre-determined order of neighborhood searches and uses a stack to perform back-tracking until it finds a path between two endpoints. Method 2 works on a graph structure which is generated from a skeleton of the artery. We used the Skan Python library [24] to extract a graph from the artery’s skeleton image. It attempts to find the shortest path from a starting node to any other node, and the shortest path between the two endpoints is found. Method 3 works on a graph structure like Method 2. It attempts to find the shortest path from a starting node to an end node via a heuristic search, which is referred to as the A* algorithm. Method 4 is the proposed method, which is described in the pseudocode of Figure 2. We counted the numbers of successful path-finding results in the left and right ICAs, respectively.

For the visual evaluation of path-finding results, we implemented a 3D visualization method that allowed for the path-finding result to be overlaid onto the segmented artery. We used the marching cubes algorithm [25] provided by the Scikit-Image Python library [26] to calculate the surface meshes of the binary arteries. For visualization, we used the Mayavi Python library (http://docs.enthought.com/mayavi/mayavi/, accessed on 27 February 2023) and created a video that plays the rotation of the 3D arterial structure along with the centerlines of the left and right ICAs.

A chi-square test was performed to find any differences between the path-finding methods in detecting the correct paths of the arterial segments. A *p*-value of <0.05 was considered statistically significant.

## 3. Results

Random shuffling of the neighbor-visiting order resulted in many possible paths being found when using the DFS algorithm. When the number of iterations was 10 in our study, there were 10 × 10 = 100 pairs of the left and right ICA paths, and the random shuffling of the neighborhood-visiting order enabled different realizations of path-finding results. It was noted that the presence of a centerline of the left or right posterior communicating artery (PComm) produced paths with significantly longer path lengths. Our experiments indicated that the overlapping paths between the left and right paths occurred only when the left and right PComm artery centerlines connected the anterior and posterior circulation arteries. This was easily handled by thresholding the path length. We empirically chose a path length of 150, which is considered to be unusually high, as the threshold. In the presence of a PComm centerline, we discarded paths whose path lengths were greater than the threshold and sought to detect a correct path among the remaining paths.

Among the 60 subjects’ data, there were 6 subjects’ data which corresponded to Case 1, 7 subject’s data corresponding to Case 2, and 47 subjects’ data corresponding to Case 3. This means that 78.3% of the data corresponded to Case 3, while 10.0% and 11.7% corresponded to Case 1 and Case 2, respectively. The proposed method identified the cases with an accuracy of 96.7%. Only two subjects’ data were misclassified. One subject’s data corresponded to Case 2 but were misclassified as Case 1. The other subjects’ data corresponded to Case 3 but were misclassified as Case 2.

The comparative accuracy results of the four path-finding methods are illustrated in Table 1. Method 4 resulted in the highest accuracy value of 95.8%, which is greater than 85.0% of Methods 1–3. As shown in Table 1, the chi-square test showed that the proposed method resulted in significantly different path-finding accuracy results when compared to the other three methods (*p* < 0.01 for the proposed method vs. DFS algorithm, *p* < 0.01 for the proposed method vs. Dijkstra algorithm, *p* < 0.01 for the proposed method vs. A* algorithm).

**Figure 9:**
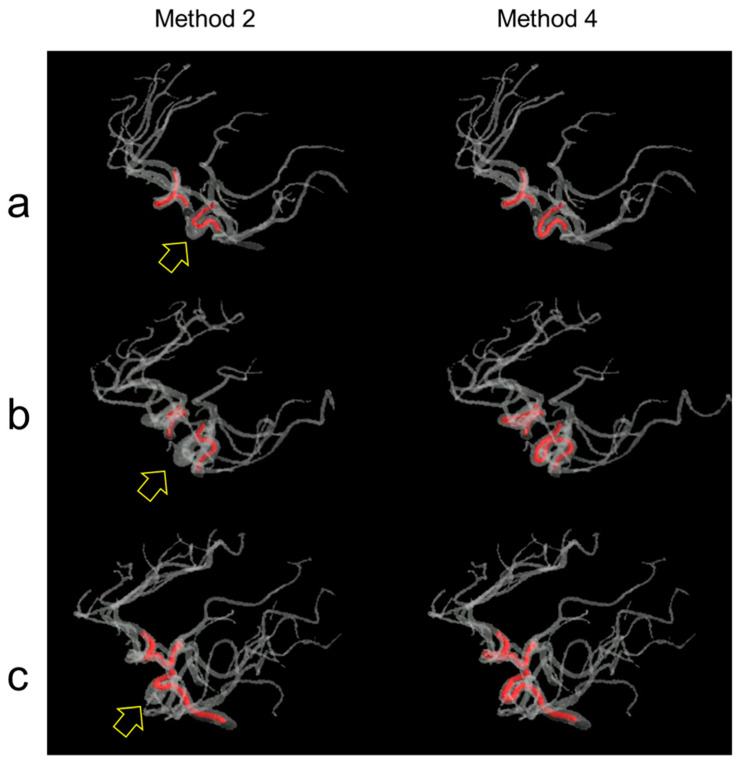
Examples of path-finding results in the ICAs of (**a**) subject A, (**b**) subject B, and (**c**) subject C. Method 2 (i.e., Dijkstra algorithm) produced incorrect path-finding results as indicated by the yellow hollow arrows, whereas Method 4 (i.e., the proposed method) produced correct path-finding results. See the videos in the Supplementary Materials.

Figure 9 shows three screenshots of the movies that play a rotated 3D visualization of the intracranial arteries, with found paths in the ICAs indicated in red. The movies are provided in the Supplementary Materials. The yellow hollow arrows indicate erroneous path-finding results with the shortest paths. Method 2 (i.e., the Dijkstra algorithm) produced incorrect paths, while Method 4 (i.e., the proposed method) produced correct paths.

**Figure 10:**
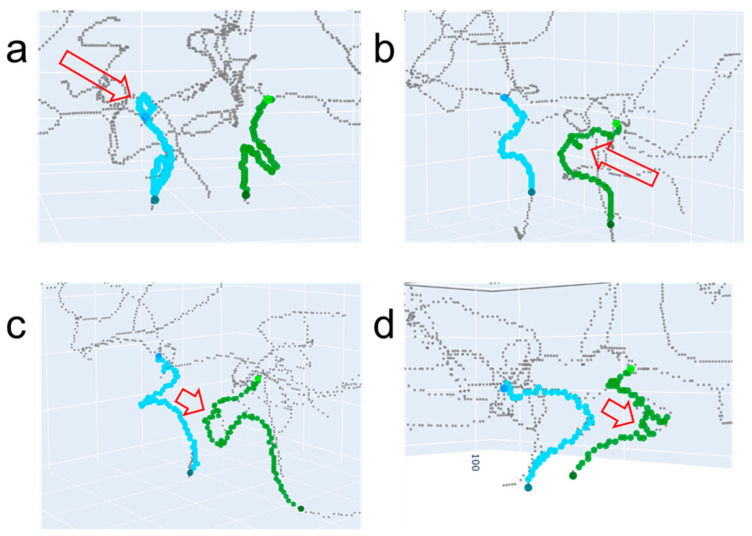
Examples of incorrect path-finding when using Method 4 (i.e., the proposed method). The red hollow arrows in (**a**–**d**) indicate regions corresponding to incorrect paths. The paths for the left ICA and right ICA are indicated by green and cyan colors, respectively.

Figure 10 shows four examples of incorrect path-finding results when using the proposed method. The regions corresponding to incorrect paths are indicated by the red hollow arrows. The ICA with an incorrect path in Figure 10a shows a small, detoured round path. Figure 10b–d show noisy non-straight paths. Notably, the incorrect paths are localized in certain regions with spurious small centerlines.

To assess the computational time of Method 4, we calculated the computational time for iterations of 10 and 20 on a Windows PC (AMD Ryzen 55,500U with Radeon Graphics 6-Core Processor Central Processing Unit). For the iteration of 10, it took approximately 1.4 s to complete the path-finding process in the ICAs. For the iteration of 20, it took approximately 2.9 s to complete the path-finding process in the ICAs.

## 4. Discussion

We demonstrated an improved path-finding algorithm for automatically identifying an artery’s centerline when two endpoints are given. The conventional Dijkstra or A* algorithms are effective in finding the shortest paths, but they may produce incorrect paths when the segmented arteries are highly tortuous such that a skeletonization process results in more than one centerline in an ICA segment. In this study, we focused on the ICA’s C4–C7 segment, which typically contains highly tortuous arterial geometry. After the skeletonization process, the segmented ICA can produce spurious centerlines, which result in erroneous paths in certain cases when using the shortest path-finding algorithms [17].

The reason why there are multiple centerlines in the tortuous ICA segment is that the 3D region growing algorithm produces over-segmented results, leading to multiple centerlines after the skeletonization process. Hence, a better segmentation method may overcome the issue of over-segmentation and avoid the situation of multiple centerlines when only one centerline contains the correct path. Simple morphological image processing such as erosion may help reduce over-segmentation, but it can remove thin arteries as a side effect. Encoder–decoder deep convolutional neural networks may have the potential to improve the segmentation results in highly tortuous arteries such as the ICA [27,28,29], and the evaluation of the centerlines after deep learning-based artery segmentation requires further investigation.

We note that the randomization of the neighbor search order is key to the generation of a variety of paths in the left and right ICAs when using the DFS-based path finding. This indeed produced almost all possible pairs of the left and right ICA paths. One can instead design predetermined neighborhood search orders, but this is not easy to implement when compared to the use of random shuffling. Notably, a recursive method that counts all available paths given the source point and the destination point also exists, but a drawback is that theoretically the recursive method has the exponential time complexity of $O(2^{V})$, where V is the number of vertices. With the various realizations of the left and right ICA paths, we developed a method that automatically chooses the correct paths by relying on the assumption that there are three possible cases of centerline compositions and by handling each case separately using the two histograms of the left and right ICAs’ path lengths.

An underlying assumption of the proposed method is that the left and right ICA segments have similar path lengths. However, in some subjects, the length of the left ICA may differ significantly from the length of the right ICA. The difference in the lengths may be related to the difference in the ranges of the lengths and could affect the choice among the three cases.

Our path-finding method is a rule-based method, and relies on the selection of the thresholds in the sum of the left and right ICA path lengths’ ranges as well as on the absolute difference in relative ranges between the left and right ICA path lengths. It may be sensitive to the choice of the thresholds. Therefore, it is worth developing a learning-based method, which would involve the development of a machine learning prediction model that takes the histogram distributions of path lengths as input and produces annotated correct paths as output.

Since the proposed method relies on a series of DFS path-finding processes with multiple realizations of neighborhood-visiting orders, it is inherently more time-consuming than the other three available methods we have considered. However, the computational time of the proposed method was not significantly longer, taking only 1.4 s per subject on a PC.

In our study, the number of ICA segments was 120 obtained from 60 subjects, which is not very large in general. Also, the majority (78.3%) of the ICAs’ centerlines corresponded to Case 3, and thus the number of incorrect paths (18) was relatively small compared to the number of all paths (120), even when using the shortest path-finding algorithms. Nonetheless, the proposed method demonstrated statistical significance in path-finding accuracy when compared with the shortest path-finding algorithms.

## 5. Conclusions

We proposed a new method that can find a path along the ICA centerline with higher accuracy than existing methods. The proposed method is based on a series of DFS algorithms where a DFS is performed after randomization of the order of visiting of neighboring voxels. Out of multiple candidate paths, appropriate paths in both left and right ICAs were identified by discarding the paths where the right ICA’s path and the left ICA’s path are overlapped and then selecting paths based on the path length distributions in the left and right ICAs. The evaluation of 60 subjects’ ICAs with the four path-finding methods shows that the proposed method outperformed the other three existing methods in path-finding accuracy. The proposed method can be useful in highly tortuous arteries such as the ICAs, in which shortest path-finding algorithms such as the Dijkstra or A* algorithms may fail to find correct paths.

## Figures and Tables

**Table 1:** Evaluation of path-finding accuracy.

		ICA		
		R	L	Total
Method 1: DFS algorithm	No. of correct paths	51	51	102
	No. of incorrect paths	9	9	18
Method 2: Dijkstra algorithm	No. of correct paths	50	52	102
	No. of incorrect paths	10	8	18
Method 3: A* algorithm	No. of correct paths	50	52	102
	No. of incorrect paths	10	8	18
Method 4: Proposed algorithm	No. of correct paths	58	57	115
	No. of incorrect paths	2	3	5
	*p*-value ^1^	-	-	0.0085
	*p*-value ^2^	-	-	0.0085
	*p*-value ^3^	-	-	0.0085

^1^ Comparison between Method 1 (DFS algorithm) and Method 4 (proposed algorithm). ^2^ Comparison between Method 2 (Dijkstra algorithm) and Method 4 (proposed algorithm). ^3^ Comparison between Method 3 (A* algorithm) and Method 4 (proposed algorithm).

**Figure A1:**
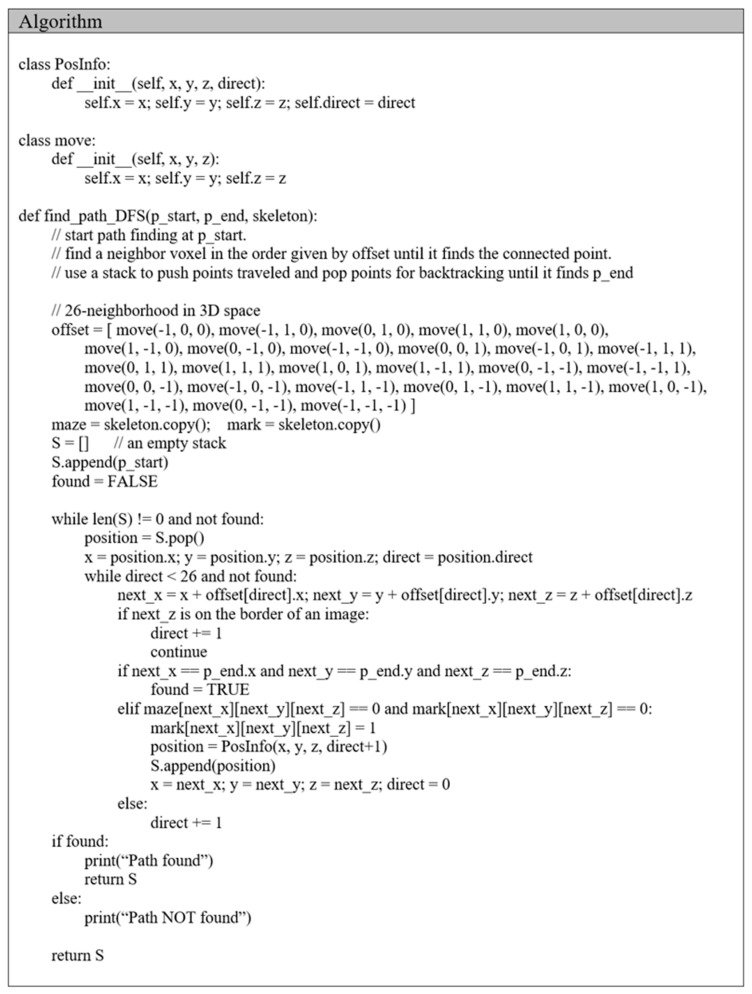
Pseudocode of the depth-first search (DFS) algorithm.

## Data Availability

The data supporting the conclusions of this article will be made available by the authors on request.

## Supplementary Materials

The supplementary videos, which compare the path-finding results between Dijkstra algorithm and the proposed method in three-dimensional rotated view, is available at: https://sites.google.com/yonsei.ac.kr/yoonckim/research/supplemental-materials (accessed on 27 February 2024).

## Appendix A

The pseudocode for the Method 1′s depth-first search (DFS) algorithm is shown in Figure A1. This is known as a solution to the maze solving problem.
